# Larone’s Medically Important Fungi: A Guide to Identification, 7^th^ Edition

**DOI:** 10.3201/eid3003.231623

**Published:** 2024-03

**Authors:** Marwan M. Azar

**Affiliations:** Yale School of Medicine, New Haven, Connecticut, USA

**Keywords:** Fungi, book review, books and media

The French chemist and microbiologist Louis Pasteur famously stated that “in the fields of observation, chance favors only the prepared mind.” How better then to be prepared for a journey into the challenging and often perplexing world of clinical mycology than with a copy of *Larone’s Medically Important Fungi* in hand? Composed with the needs of the medical mycology technician in mind (which, fortunately, translate equally well to the needs of laboratorians, physicians, and trainees alike) and written in the style of a field guide to identification, Larone’s guide serves as an easily accessible yet surprisingly granular compendium of medically important fungi (Figure).

**Figure:**
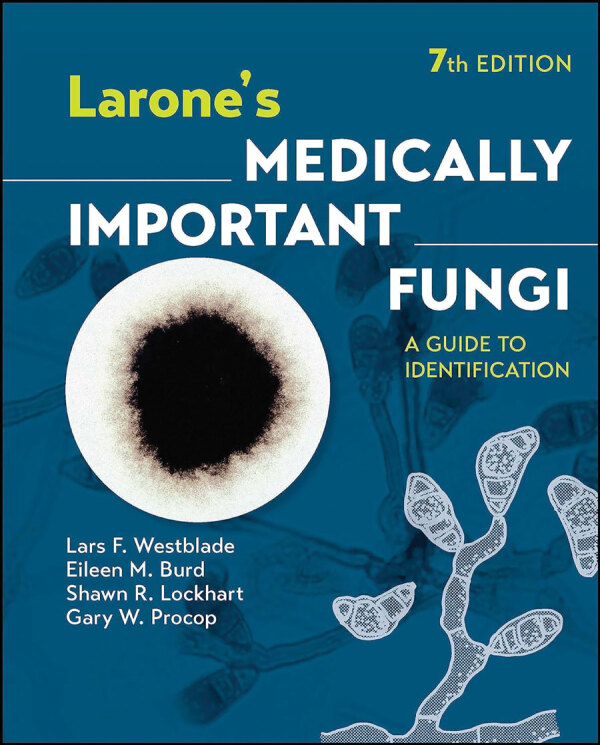
Larone’s Medically Important Fungi: A Guide to Identification

 As stated in the book’s preface, this manual does not include an exhaustive account of the epidemiology, pathophysiology, diagnosis, and therapeutics for each fungal pathogen, nor is it designed to replace more comprehensive mycology textbooks. There are other well-known and exhaustive references for that. The aim of this guidebook is to help provide a rapid preliminary diagnosis within the trenches of the microbiology laboratory, based only on the colony and microscopic morphology of a cultured organism or, at times, its morphology on direct stains. 

In keeping with this purpose, this newest (7th) edition’s format adheres to an ergonomic design, with a color-coded layout that increases its usability in real time. The book begins with a Basics section to orient readers on how to wield the guide and ends with a highly useful image appendix and glossary of commonly used, but sometimes nebulous, terms (e.g., blastoconidium, the technical term for a unicellular yeast). The Basics section begins ominously, by cautioning that readers “should understand several points” before using the guide; such counsel is justified, given that the practice of fungi identification in the medical setting is highly nuanced and requires strict adherence to standard laboratory procedures of quality and safety for favorable results. The meat of the matter lies in the middle of the book, which features 4 core sections on direct identification of fungi from clinical specimens, identification of fungi from cultured isolates, basics of molecular methods of fungal identification, and laboratory techniques. The first 2 sections stand out as outstanding compendia of clinically important fungi, with pithy descriptions of each pathogen, its taxonomy, pathogenicity, site of infection, accompanying tissue reactions, and microscopic and colony morphologies. Each fungi discussion is accompanied by a hand-drawn sketch of the organism’s distinct morphology alongside one or more representative photomicrographs. Many of the photos are in color, but a large number are unfortunately monochrome, making them less visually appealing and rendering the depicted structures harder to discern. A full-color image appendix partially makes up for this concern. 

Importantly, most organisms are arranged according to their morphological similarities rather than alphabetically to make comparisons between similarly appearing structures easier. This categorization works well with the included discussion of nonfungal pathogens (e.g., actinomycetes and *Prototheca*), which closely resemble fungi microscopically.

One of the reasons Larone’s guide is such an effective mycology handbook is because it takes nothing for granted. Replete with explanations of basic histological terms, ranging from abscess to Splendore-Hoeppli phenomenon, and descriptions of fundamental tissue reactions to fungal infection (e.g., granulomatous inflammation)—all complemented by helpful summary tables and explanatory figures—the book achieves the remarkable feat of being simultaneously concise and complete. Although the emphasis is on usability, readers will enjoy the breadth of information provided. The book is well edited, and the newest edition now includes information on emerging pathogens, such as *Emergomyces* and *Emmonsia* species.

Larone’s guide is not meant to be the sort of book one peruses cover to cover nor the subject of a leisurely read, but the kind of book that is never far away from the bench, the microscope, or the office. It appeals to all levels of expertise—from mycologists-in-training to seasoned experts and from academic to commercial laboratories—because it provides the actionable information needed to make a diagnosis. I was gifted a copy of an earlier edition as a budding clinical mycologist and have since reached for it countless times. Joining the pantheon of revered medical tomes is no small feat, yet Larone’s guide successful formula has enabled it to accomplish just that.

