# Correction: Vol. 30, No. 4

**DOI:** 10.3201/eid3004.C13004

**Published:** 2024-04

**Keywords:** errata, erratum, correction

The author list was incorrect in Larone’s Medically Important Fungi: A Guide to Identification, 7th Edition (M.M. Azar). The article has been corrected online (https://wwwnc.cdc.gov/eid/article/30/3/23-1623_article).

